# Managing emergencies: lessons from aviation

**Published:** 2018-11-09

**Authors:** David Yorston, Mike Duncalfe

**Affiliations:** 1 Consultant Ophthalmologist: Tennent Institute of Ophthalmology, Gartnavel Hospital, Glasgow, Scotland, UK.; 2 Flight Operations Manager: Mission Aviation Fellowship, Papua New Guinea.

**There is no need to be surprised by an eye emergency – preparation and practice make all the difference.**

Many will remember the remarkable pictures and story of the US Airways passenger jet that lost all power and landed on the Hudson river, in the middle of New York, without loss of life or serious injuries. Although emergencies are rare in the day to day routine of modern air transport, the aviation industry still devotes a great deal of time and money to learning how to avoid accidents and emergencies, and also how to handle them when they happen.

Much has been written and said about the parallels and differences between aviation and medicine. Although an eye clinic is very different from an aircraft, there are things we can learn from the air transport industry's approach to managing emergencies.

Airlines and aircraft manufacturers devote considerable time and resources to planning for emergencies. As flying in a commercial aircraft is now the safest way to travel per passenger mile, aviation emergencies are increasingly rare. Despite this, preparedness for emergencies continues to be a priority for the industry. Although the ophthalmic emergencies discussed in this issue of the journal are all, individually, relatively uncommon, all eye workers will at some point encounter patients in need of emergency treatment. The outcomes for these patients – whether or not they regain their sight – will depend on the time and effort that eye health workers put in preparing for such emergencies.

## Teamwork

The aviation industry's culture of preparedness is based on crew resource management (CRM).^1^ In summary, CRM is an approach that requires the whole team to be prepared, not just the pilot. If an emergency occurs, every member of the crew has a role in dealing with it, and each member of the crew carries that responsibility. In the setting of an eye clinic, this means that management of an emergency is not just the job of the ophthalmologist, but of the whole team working together. For example, if a patient attends with a severe corneal ulcer, the clinic receptionist should recognise that this is a serious problem, and ensure that they are seen promptly. The eye nurse identifies that this is probably a bad corneal ulcer, and ensures that the equipment needed to take a specimen is ready and available. The pharmacist can start to prepare high potency eye drops so that treatment can be started as soon as the diagnosis is confirmed. The ophthalmologist listens to the input of the nurses and other eye clinic workers, so that she or he is ready to take the specimens and start the treatment.

## Standard operating procedures

Aviation also relies on having standard operating procedures in place. These are written guidelines and protocols that give details about what action should be taken in the event of an emergency. Although you may think you know how to manage acute glaucoma, having it written down, and accessible, minimises the risk of making a mistake or forgetting something. All eye clinics should have written protocols for eye emergencies. These should be written for the clinic, and give specific instructions, e.g., a list of the equipment needed to take a specimen from an infected corneal ulcer, or a description of how to prepare the correct dose of antibiotics for intravitreal injection. All eye clinic personnel should have access to the protocols at all times.

**Figure:**
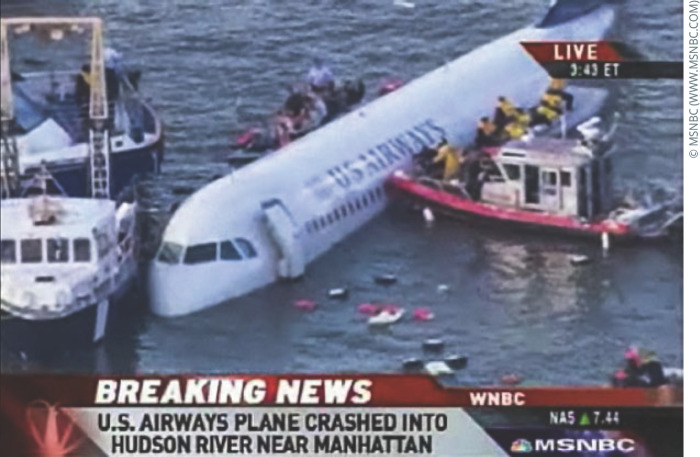
Planning and preparation helped to avoid loss of life when US Airways Flight 1549 crash-landed on the Hudson river, New York, in 2009. USA

## Preparation and practice

Air crews practise and simulate emergencies. Pilots have access to complex and expensive simulators that allow them to experience what it is like to fly an aircraft after an engine failure. When the real event occurs, their training and experience help them to make the correct decisions. In ophthalmology we don't need complicated simulators to prepare for emergencies. Teams can practise preparing intravitreal antibiotics with a few syringes. The theatre team can prepare for the management of vitreous loss by keeping a vitreous cutter aside as a practice instrument and carrying out regular drills to ensure that all the theatre nurses know how to assemble and connect it.

Everyone needs to devote time and energy to planning and preparing for emergencies. The training and preparation has to extend to the whole eye care team so that everyone understands their responsibilities, and, with the aid of standard guidelines, knows exactly what they need to do when confronted by a patient with an ophthalmic emergency.
